# Current Hospital Topics

**Published:** 1906-08-04

**Authors:** 


### HOSPITAL ADMINISTRATION.

### CONSTRUCTION AND ECONOMICS.

### CURRENT HOSPITAL TOPICS.

#### King Edward's Hospital Fund.

MR. MAYNARD, who took high honours at Oxford, and has devoted himself to the study of economics, after spending a few years in the secretariat of various movements having for their object the relief of the unemployed, has been appointed Secretary to the King's Hospital Fund. Mr. Maynard's record shows him to be a man of considerable attainments, and we hope that his appointment may prove of real benefit to the many and important interests which the King's Fund represents.

#### Royal Hospital for Incurables.

MR. CHARLES CUTTING, who has been Assistant Secretary and Steward at the Royal Free Hospital for ten and a half years, has been appointed Secretary of this institution. Mr. Cutting commenced his work as an assistant in Dr. Barnardo's offices, and he has contributed occasionally to magazines and newspapers. He obtained a prize in 1903 for the best story suitable for reading aloud to children, and our readers may remember some contributions of his entitled "Hospital Secrets" which were published in a section of THE HOSPITAL some years ago. A hospital for incurables is a very difficult establishment to adminster justly and efficiently. We have always felt that the Royal Hospital for Incurables at Putney needed a strong hand to overhaul the whole administration, to soften and modernise it, and so to add greatly to the efficiency and popularity of this institution.

#### The Finances of County Hospitals.

SEVERAL of the county hospitals are finding that the increased expense of conducting a great modern hospital renders it essential that each county hospital shall reorganise its methods of raising money with a view to induce a much larger number of people than formerly to associate themselves with the work by becoming annual subscribers. In the county of Salop a few years ago the Shrewsbury Infirmary authorities set to work to arouse the county by village and district meetings, and the appointment of representatives in each centre of population which sends patients to the hospital. The financial result was eminently satisfactory. The Norfolk and Norwich Hospital has determined to appoint committees in various parts of the county with the view to enlist the services of people to represent the hospital in various districts, and to bring its objects and needs more prominently before those who can afford to contribute to its funds. Patients are received from all parts of the county of Norfolk, and it is only reasonable that adequate subscriptions should be obtained from each district which contributes patients. The Shrewsbury experiment should encourage Sir Charles Gilman and the committee to lose no time in organising a series of meetings throughout the county of Norfolk in support of the Norfolk and Norwich Hospital. We wish them all success in this new departure, which proves that the management of one of the most reputable and valuable of county hospitals is to-day in able hands.

#### Isolation Hospital Extravagance.

IN our issue of May 12 we called attention to an inquiry by the Local Government Board Inspector into the expenditure of the Joint Isolation Hospital, Thistle Hill, near Knaresborough. Dr. Johnstone, the inspector, has now reported, and the Local Government Board have written to the Town Clerk to point out that the hospital has been furnished in so elaborate a fashion that the general result has been to bring the cost of the furniture to over £53 per bed, a figure greatly in excess of the average cost of the furnishing of similar hospitals in other places. The Board then drew attention to certain items of extravagance, to which we have already called attention, and they emphasise their finding by the statement that experience has shown that £35 per bed is amply sufficient to provide good, substantial, and suitable furniture for an isolation hospital, if the furnishing is carried out in a reasonable and economical manner. On the basis of £35 per bed the furnishing of the Joint Board's hospital with forty-eight beds should have amounted to £1,680; in fact, the cost was £2,561, and the Local Government Board have surcharged the local authority with the difference—namely, £881. On receipt of this communication the Board resolved to provide the £881 out of revenue account—a fact which, we hope, the ratepayers concerned will make a note of. It would seem from the discussion at the last meeting of the Dewsbury Joint Hospital Board that the ratepayers must arouse themselves and take steps to select as their representatives men of business who will treat the management of this hospital on business lines. What, for instance, can be said in defence of a decision which the Board has arrived at, to charge each authority 17s. 6d. as the cost of each fever patient, when it was admitted in evidence that the actual cost exceeds £1 per week? We are glad to note that the Heckmondwike representatives voted against this charge as unsatisfactory. We beg to call the attention of the local press and the ratepayers to the proceedings of this Board, with the view to the introduction of necessary reforms into the management of the affairs of this isolation hospital with as little delay as possible.

